# Distinct Components in the Right Extended Frontal Aslant Tract Mediate Language and Working Memory Performance: A Tractography-Informed VBM Study

**DOI:** 10.3389/fnana.2020.00021

**Published:** 2020-04-21

**Authors:** Federico Varriano, Saül Pascual-Diaz, Alberto Prats-Galino

**Affiliations:** Laboratory of Surgical Neuroanatomy, University of Barcelona, Barcelona, Spain

**Keywords:** frontal aslant tract, extended frontal aslant tract, FAT, exFAT, voxel-based morphometry, working memory, language, HCP

## Abstract

The extended frontal aslant tract (exFAT) is a tractography-based extension of the frontal aslant tract (FAT) which has been shown to be related with language and working memory performance in healthy human adults, but whether those functional implications map to structurally separate regions along its trajectory is still an open question. We present a tractography-informed Voxel-Based Morphometry procedure capable of detecting local tract-specific structural differences in white matter regions and apply it in two maximum variation sampling studies by comparing local differences in diffusion-derived microstructural parameters and fiber density along the exFAT territory between top performers and bottom performers in language and working memory tasks. In the right hemisphere we were able to detect, without prior constraints, a vertical frontal aslant component approximating the original FAT trajectory whose fiber density was significantly correlated with language (but not working memory) performance and an anterior cluster component corresponding to a distinct anterior frontal aslant component whose fiber density was significantly correlated with working memory (but not language) performance. The reported sub-division of the exFAT territory describes a set of frontal connections that are compatible with previously reported results on the Broca’s territory and frontal cortex hierarchical organization along an anterior-posterior gradient, suggesting that the exFAT could be part of a common neuroanatomical scaffold where language and working memory functions are integrated in the healthy human brain.

## Introduction

The frontal aslant tract (FAT) is a recently discovered left-lateralized bilateral tract described in virtual dissection studies (Catani et al., 2012) as a connection between Broca’s territory and the supplementary and pre-supplementary motor areas of the superior frontal gyrus (SFG). The FAT has also been identified in post-mortem dissection using Klinger’s technique, with a good match between the dissected tract and tractography reconstructions (Vergani et al., 2014; Bozkurt et al., 2016), and an homologous tract has been described in monkeys (Thiebaut de Schotten et al., 2012).

The microstructural properties of the bilateral FAT has been correlated with visually guided hand movements (Budisavljevic et al., 2016), and its damage predicted speech fluency alterations in chronic post-stroke aphasia (Basilakos et al., 2014). Furthermore, ample evidence from functional connectivity disruptions induced by intraoperative electrical stimulation during awake surgery suggests that the left FAT is implicated in language function, with transient speech arrest (Vassal et al., 2014; Fujii et al., 2015), alteration of morphological derivation rules in speech production (Sierpowska et al., 2015), transient post-operative speech initiation disorders (Kinoshita et al., 2014) and mutism that persisted 5 days after the operation but was resolved after 3 months (Kemerdere et al., 2016). An extensive review of the FAT implication in speech, language and executive function reports an emerging consensus of a left FAT specialization for speech actions, and a right FAT specialization for executive control mediated by inhibitory control (Dick et al., 2018).

Several diffusion-derived parameters, such as fractional anisotropy (FA) and other simpler lambda-derived diffusivity parameters, such as axial diffusivity (AD), radial diffusivity (RD) and mean diffusivity (MD), have been used to study the structural properties of the FAT territory: in the seminal article approaching the tractography study of the FAT (Catani et al., 2013), FA and RD were used as surrogate measures of white matter spatial organization, myelination and axonal integrity, and were found to be correlated with mean length of utterance and words per minute scores in patients with primary progressive aphasia (PPA). In a different study, complementary results were reported showing that the microstructural properties of the FAT in patients with persistent developmental stuttering, as measured by lambda diffusivity indices, was related to speech production. Specifically, a significant group difference in the average MD of the entire tract was detected (Kronfeld-Duenias et al., 2016). In this line, another study focused on the FA values in the FAT in patients with PPA, arguing that the lambda diffusivity values might introduce systematic biases, and found significant correlations between longitudinal white matter changes and FA in the left FAT (Mandelli et al., 2016). From a development perspective, a study with children between ages 5 and 8 showed that the microstructural properties of the FAT, as measured by AD, RD, MD and FA, remained mostly stable (Broce et al., 2015), although a later study with a larger sample and more powerful methods showed that the FAT presents changes in anisotropy measures until adulthood, and that microstructural properties of the right FAT are associated with increased reports of attention problems in children (Garic et al., 2018).

The extended FAT (exFAT) has been proposed as an extension of the original FAT territory allowing for unrestricted connectivity between Broca’s territory and the totality of the SFG regions of interest (ROIs), and its volume has been shown to correlate both with language function (bilaterally) and with working memory capacity (in the right hemisphere) (Varriano et al., 2018). These mixed-function results are consistent with the current knowledge that working memory function [and general intelligence, which is closely related (Colom et al., 2007)] is dependent on frontal lobe structural parameters as established by evidence from volumetric studies (Haier et al., 2004), FA studies (Nagy et al., 2004) and tractography studies (Darki and Klingberg, 2015), and is affected by frontal lesions (Boisgueheneuc et al., 2006; Szczepanski and Knight, 2014).

Related to the mixed working memory and language function implication of the right exFAT, it has been reported that resections of the right prefrontal region in glioma surgery induced significant chronic spatial working memory deficits without motor and language alteration (Kinoshita et al., 2016), and it has been reported that stroke-induced damage to the right inferior frontal sulcus can result in long lasting speech comprehension impairments in right-handed subjects without atypical language lateralization (Gajardo-Vidal et al., 2018).

Although several influential theoretical models approach working memory and language as independent systems [e.g., in the works of Baddeley and Hitch (Baddeley, 2003)] it has been proposed that working memory and language function could present overlapping neural correlates (Jacquemot and Scott, 2006) and share a common neural substrate (Acheson et al., 2011; Emmorey et al., 2017). In young children, it has been reported that separate but interacting components of working memory can be distinguished by the roles they play in supporting language acquisition (Engel de Abreu et al., 2011). From a neuroanatomical point of view, brain connectivity can be studied from a number of parameter- and scale-dependant approaches that offer complementary descriptions (Goulas et al., 2014). In the frontal lobe, it has been proposed that both Broca’s area (and its non-language dominant homolog contralateral region) (Koechlin and Jubault, 2006) and the dorsal region of the frontal cortex (Badre and D’Esposito, 2009) could be organized along a rostro-caudal gradient where more anterior regions are implicated in more abstract/higher order mental functions. Here, we present a robust tract-specific methodology based on the voxel-based morphometry (VBM) technique (Ashburner and Friston, 2000) and use it to investigate the possibility that the exFAT territory could be part of a neuroanatomical scaffold that integrates working memory and language function in the human brain.

Voxel-based morphometry is a powerful and well-established technique that can be employed to detect local differences in concentration and density of brain tissue through a voxel-wise comparison of multiple registered brain images. VBM has been widely applied to the study of normal brain development, structural differences between different populations and morphological alterations in a large number of clinical conditions. Although VBM studies usually focus on the analysis of gray matter territories, this technique has also been successfully applied to the investigation of the white matter regions (Good et al., 2002; Chaim et al., 2007; Golestani et al., 2007; Wang et al., 2010) and a wide range of meta-analytic evidence is available in the literature to this regard (Honea et al., 2005; Li et al., 2012; Ganzola and Duchesne, 2017; Pezzoli et al., 2018). In the present study we propose the application of a VBM pipeline along the exFAT white matter territory to study local differences in fiber density and diffusion-derived microstructural parameters such as FA, AD, RD, and MD among healthy subjects.

### Hypotheses

Given the presented knowledge on frontal structures and aslant connections, we posit that the exFAT territory can be subdivided into a vertical component corresponding to the original FAT territory, and therefore having a well-described language function implication, and a previously unreported anterior frontal aslant component (the aFAT) connecting the aforementioned high order regions, which would thus show working memory performance implications.

Specifically, within the context of a VBM study performed by comparing local differences in fiber densities along the exFAT territory, we state the following hypotheses:

(a) Maximum variation sampling by language performance will yield a significant bilateral FAT component, whose average fiber density will be significantly correlated with language performance.
(b) Maximum variation sampling by working memory performance will yield a significant right aFAT component, whose average fiber density will be significantly correlated with working memory performance.
(c) Maximum variation sampling by working memory performance will not detect any significant component in the left hemisphere.

## Materials and Methods

Four groups of 35 healthy subjects each were drawn from the HCP900 data release (S900) using maximum variation sampling by selecting 35 bottom performers and 35 top performers in the HCP language task (Binder et al., 2011), and 35 bottom performers and 35 top performers in general scores for the 2-back working memory task of the HCP dataset. Raw scores in the language task were weighted by task difficulty in order to increase its discriminative power.

For each subject, a tractography reconstruction of the bilateral exFAT was performed using MRTrix3 (Tournier et al., 2012) with iFOD2 (Tournier et al., 2010) following a reported methodological procedure for exFAT reconstruction (Varriano et al., 2018). A total of 5 million fibers per tract were fired and density maps of the exFAT territory were built for each tract.

We built a VBM pipeline using FSL (Jenkinson et al., 2012) and applied it to the four experimental groups in two study designs to compare fiber densities along the exFAT territory with performance scores in top performers vs. bottom performers as follows.

A common MNI152 template was created for each study design by iteratively aligning and warping each subject’s white matter parcelation to the common FSL Standard White Matter Tissue prior template with a rigid first pass registration and a non-linear second pass registration. Jacobian determinants were calculated to account for absolute differences in white matter volume. Then, using the calculated transformations, each corresponding exFAT territory density map was warped to the common template and Jacobian modulation was performed. A 2 mm Gaussian kernel was applied to the resulting volumes, which will be referred to as territory-informed regions for VBM analysis.

For the working memory extreme groups, we created (a) a correlation design between diffusion-derived parameters along the bilateral exFAT territory voxels and the scores in the general 2-back task, while controlling for age, total brain volume (O’Brien et al., 2011) and scores in the control 0-back task, and (b) a two-sample unpaired *t*-test design to assess whether diffusion-derived parameters differences in specific voxels of the exFAT territory explained the difference of scores in the 2-back task between high performers and low performers populations, while modeling the effects of age, total brain volume and baseline 0-back scores as confounds.

For the language extreme groups, we created (a) a correlation design between diffusion-derived parameters along the bilateral exFAT territory voxels and the scores in the adjusted language task, while controlling for handedness, age and total brain volume, and (b) a two-sample unpaired *t*-test design to assess whether diffusion-derived parameters differences in specific voxels of the exFAT territory explained the difference of scores in the adjusted language task between high performers and low performers populations, modeling the effects of handedness, age and total brain volume as confounds.

Each experimental design was used to execute a non-parametric permutation inference procedure [the “randomize” algorithm (Winkler et al., 2014)] along left and right exFAT territories with 10^4^ permutations. Variables were demeaned, threshold-free cluster enhancement (TFCE) (Smith and Nichols, 2009) was applied and a variance smoothing parameter of 2 mm was used. In order to filter artifactual results, and increase the sensitivity, clusters containing less than 10 voxels were discarded, if present, following standard practice (Radua et al., 2012; Radua Joaquim et al., 2014; Patil et al., 2017; Tench et al., 2017).

This general procedure for detection of local differences in structural parameters was applied to FA, AD, RD, MD and fiber density images along the exFAT territory.

For the fiber density cases, the resulting significant clusters were then warped back to each subject’s native space using inverse transforms and were used as inclusion ROIs to filter the original exFAT tractography reconstructions. The selected fibers were then used to construct new density maps that extend the territory-informed clusters by taking into account the directional and structural information present in the white matter, as captured by the advanced tractography algorithm. These new density maps will thus be referred to as tractography-informed regions for VBM analysis. The non-parametric permutation inference procedure was then repeated for the tractography-informed exFAT regions to evaluate whether the inclusion of tractography-derived structural information into the clusters resulted in an improved detection of distinct frontal aslant components.

Reported *p*-values correspond to an adjusted significance level of α = 0.05 after controlling for the family-wise error rate (FWER) (Nichols and Holmes, 2002), providing a conservative correction of the multiple comparison problem.

**FIGURE 1:**
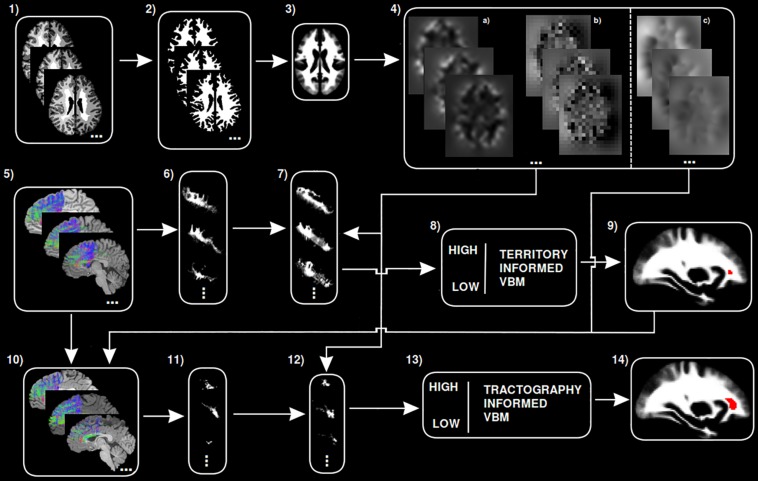
Step-by-step graphical representation of the territory-informed and tractography-informed VBM procedures. (1) Original brain parcelations. (2) Extracted white matter segmentations. (3) Study template. (4) (a) Jacobian images for modulation, (b) Warp maps, (c) Inverse warp maps. (5) Original exFAT tractography. (6) Original exFAT density maps in native space. (7) Original exFAT density maps in template space, modulated. (8) VBM analysis along the exFAT territory. (9) Significant cluster for territory-informed VBM shown in red. (10) Tractography-informed exFATs. (11) Tractography-informed density maps in native space. (12) Tractography-informed density maps in template space, modulated. (13) VBM analysis along the tractography-informed exFAT region. (14) Significant cluster for tractography-informed VBM shown in red.

A step by step graphical description of the tractography-informed VBM procedure to investigate local differences in fiber density along the exFAT territory can be seen in Figure 1.

This study was conducted with the approval of the Bioethics Committee of the University of Barcelona. Institutional Review Board (IRB00003099).

## Results

### AD Study

The AD-based VBM study detected, in the right hemisphere, a significant cluster located in the posterior region of the exFAT territory in the language extreme group for both the correlation test (*p* = 0.012) and the unpaired *t*-test (*p* = 0.015). Results are shown in Table 1 and Supplementary Figures 1, 2.

**TABLE 1:** AD VBM results for maximum variation sampling groups.

AD VBM		Cluster size	Mean adj *p*-val (SD)	Min robust adj *p*-val	X pMin	Y pMin	Z pMin
Language right	Correlations	256	0.012(0.013)	0.0016	26	63	46
	*t*-Tests	219	0.015(0.014)	0.0025	26	63	46
Language left	Correlations	–	–	–	–	–	–
	*t*-Tests	–	–	–	–	–	–
Working memory right	Correlations	–	–	–	–	–	–
	*t*-Tests	–	–	–	–	–	–
Working memory left	Correlations	–	–	–	–	–	–
	*t*-Tests	–	–	–	–	–	–

*Cluster size in voxels. Mean adj *p*-val (SD) indicates FWE-corrected *p*-values and standard deviation. Min robust adj *p*-val indicates the robust minimum FWE-corrected *p*-value (approximately 2nd percentile). (X pMin, Y pMin, Z pMin) indicate the coordinates of the voxel with the smallest FWE-corrected *p*-value in MNI152 space.*

### RD Study

The RD-based VBM study detected, in the right hemisphere, a significant cluster located in the posterior region of the exFAT territory in the language extreme group for both the correlation test (*p* = 0.014) and the unpaired *t*-test (*p* = 0.016). Results are shown in Table 2 and Supplementary Figures 3, 4.

**TABLE 2:** RD VBM results for maximum variation sampling groups.

RD VBM		Cluster size	Mean adj *p*-val (SD)	Min robust adj *p*-val	X pMin	Y pMin	Z pMin
Language right	Correlations	141	0.014(0.0138)	0.0017	27	63	47
	*t*-Tests	128	0.016(0.0149)	0.0026	27	63	48
Language left	Correlations	–	–	–	–	–	–
	*t*-Tests	–	–	–	–	–	–
Working memory right	Correlations	–	–	–	–	–	–
	*t*-Tests	–	–	–	–	–	–
Working memory left	Correlations	–	–	–	–	–	–
	*t*-Tests	–	–	–	–	–	–

*Cluster size in voxels. Mean adj *p*-val (SD) indicates FWE-corrected *p*-values and standard deviation. Min robust adj *p*-val indicates the robust minimum FWE-corrected *p*-value (approximately 2nd percentile). (X pMin, Y pMin, Z pMin) indicate the coordinates of the voxel with the smallest FWE-corrected *p*-value in MNI152 space.*

### MD Study

The MD-based VBM study detected, in the right hemisphere, a significant cluster located in the posterior region of the exFAT territory in the language extreme group for both the correlation test (*p* = 0.013) and the unpaired *t*-test (*p* = 0.015). Results are shown in Table 3 and Supplementary Figures 5, 6.

**TABLE 3:** MD VBM results for maximum variation sampling groups.

MD VBM		Cluster size	Mean adj *p*-val (SD)	Min robust adj *p*-val	X pMin	Y pMin	Z pMin
Language right	Correlations	216	0.013(0.0134)	0.0015	27	62	47
	*t*-Tests	186	0.015(0.0149)	0.0022	27	62	47
Language left	Correlations	–	–	–	–	–	–
	*t-*Tests	–	–	–	–	–	–
Working memory right	Correlations	–	–	–	–	–	–
	*t*-Tests	–	–	–	–	–	–
Working memory left	Correlations	–	–	–	–	–	–
	*t*-Tests	–	–	–	–	–	–

*Cluster size in voxels. Mean adj *p*-val (SD) indicates FWE-corrected *p*-values and standard deviation. Min robust adj *p*-val indicates the robust minimum FWE-corrected *p*-value (approximately 2nd percentile). (X pMin, Y pMin, Z pMin) indicate the coordinates of the voxel with the smallest FWE-corrected *p*-value in MNI152 space.*

### FA Study

The FA-based VBM study detected, in the right hemisphere, a significant cluster located in the posterior region of the exFAT territory in the language extreme group for both the correlation test (*p* = 0.015) and the unpaired *t*-test (*p* = 0.016). In addition, a significant cluster located in the anterior region of the right exFAT territory was detected in the working memory extreme group for the correlation test (*p* = 0.041). Results are shown in Table 4 and Supplementary Figures 7–9.

**TABLE 4:** FA VBM results for maximum variation sampling groups.

FA VBM		Cluster size	Mean adj *p*-val (SD)	Min robust adj *p*-val	X pMin	Y pMin	Z pMin
Language right	Correlations	263	0.015(0.0157)	0.0016	26	63	46
	*t*-Tests	196	0.016(0.0148)	0.0022	26	63	46
Language left	Correlations	–	–	–	–	–	–
	*t*-Tests	–	–	–	–	–	–
Working memory right	Correlations	11	0.041(0.0053)	0.0329	29	81	36
	*t*-Tests	–	–	–	–	–	–
Working memory left	Correlations	–	–	–	–	–	–
	*t*-Tests	–	–	–	–	–	–

*Cluster size in voxels. Mean adj *p*-val (SD) indicates FWE-corrected *p*-values and standard deviation. Min robust adj *p*-val indicates the robust minimum FWE-corrected *p*-value (approximately 2nd percentile). (X pMin, Y pMin, Z pMin) indicate the coordinates of the voxel with the smallest FWE-corrected *p*-value in MNI152 space.*

### Fiber Density Study

Both the territory-informed and the tractography-informed procedures yielded distinct significant clusters that were separated antero-posteriorly along the right exFAT territory. No significant clusters were found for the left exFAT territory in any study design.

The territory-informed clustering procedure detected, in the right hemisphere, a significant cluster located in the posterior region of the exFAT territory in the language extreme group for both the correlation test (*p* = 0.032) and the unpaired *t*-test (*p* = 0.042), and a significant cluster located in the anterior region of the exFAT territory in the working memory extreme group for both correlation test (*p* = 0.026) and the unpaired *t*-test (*p* = 0.024). Results for the territory-informed clustering procedure are shown in Table 5.

**TABLE 5:** Territory-informed VBM results for maximum variation sampling groups.

Territory-informed VBM		Cluster size	Mean adj *p*-val (SD)	Min robust adj *p*-val	X pMin	Y pMin	Z pMin
Language right	Correlations	30	0.032(0.0074)	0.02	26	65	46
	*t*-Tests	14	0.042(0.00584)	0.033	25	65	46
Language left	Correlations	–	–	–	–	–	–
	*t*-Tests	–	–	–	–	–	–
Working memory right	Correlations	50	0.026(0.0124)	0.009	28	82	37
	*t*-Tests	44	0.024(0.0106)	0.0087	28	82	37
Working memory left	Correlations	–	–	–	–	–	–
	*t*-Tests	–	–	–	–	–	–

*Cluster size in voxels. Mean adj *p*-val (SD) indicates FWE-corrected *p*-values and standard deviation. Min robust adj *p*-val indicates the robust minimum FWE-corrected *p*-value (approximately 2nd percentile). (X pMin, Y pMin, Z pMin) indicate the coordinates of the voxel with the smallest FWE-corrected *p*-value in MNI152 space.*

The tractography-informed clustering procedure detected, in the right hemisphere, significant clusters located in the posterior region of the exFAT territory in the language extreme group for both the correlation test (*p* = 0.009) and the unpaired *t*-test (*p* = 0.01), and significant clusters located in the anterior region of the exFAT territory in the working memory extreme group for both correlation test (*p* = 0.007) and the unpaired *t*-test (*p* = 0.007). Results for the tractography-informed clustering procedure can be seen in Table 6.

**TABLE 6:** Tractography-informed VBM results for maximum variation sampling groups.

Tractography-informed VBM		Cluster size	Mean adj *p-*val (SD)	Min robust adj *p*-val	X pMin	Y pMin	Z pMin
Language right	Correlations	407	0.009(0.0126)	0.001	25	65	45
	*t*-Tests	366	0.01(0.0134)	0.001	25	65	46
Language left	Correlations	–	–	–	–	–	–
	*t*-Tests	–	–	–	–	–	–
Working memory right	Correlations	334	0.007(0.0096)	0.001	26	82	36
	*t*-Tests	338	0.007(0.0097)	0.001	27	82	36
Working memory left	Correlations	–	–	–	–	–	–
	*t*-Tests	–	–	–	–	–	–

*Cluster size in voxels. Mean adj *p*-val (SD) indicates FWE-corrected *p*-values and standard deviation. Min robust adj *p*-val indicates the robust minimum FWE-corrected *p*-value (approximately 2nd percentile). (X pMin, Y pMin, Z pMin) indicate the coordinates of the voxel with the smallest FWE-corrected *p*-value in MNI152 space.*

Compared to the territory-informed clusters, the tractography-informed clusters showed a very high coordinate proximity for the peak significance voxel, larger cluster sizes, and extended the results along the aslant trajectory of the exFAT fibers, delineating a vertical component approximating the original FAT trajectory and a previously unreported anterior frontal aslant trajectory.

**FIGURE 2:**
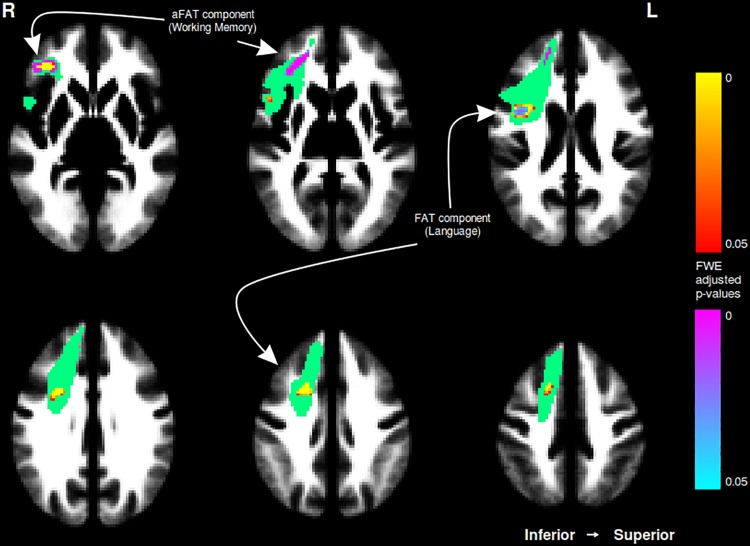
Axial cuts depicting the right exFAT territory in green. Territory-informed aFAT component shown in hot colormap, tractography-informed aFAT component shown in cool colormap. Territory-informed FAT component shown in cool colormap, tractography-informed FAT component shown in hot colormap. A set of labels and arrows point to specific examples where the aFAT and FAT components can be clearly identified.

**FIGURE 3:**
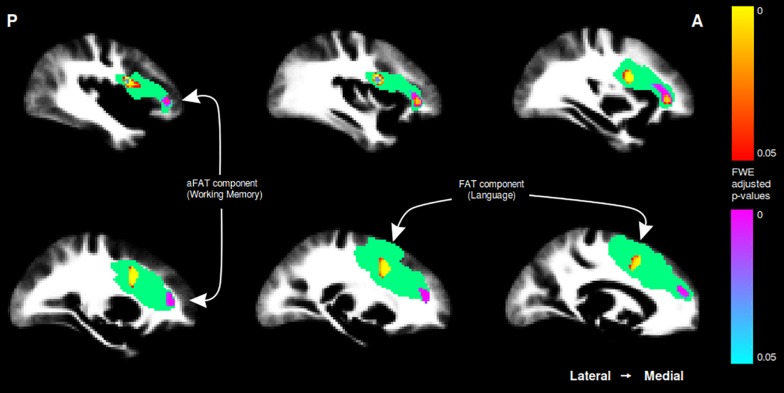
Sagittal cuts depicting the right exFAT territory in green. Territory-informed aFAT component shown in hot colormap, tractography-informed aFAT component shown in cool colormap Territory-informed FAT component shown in cool colormap, tractography-informed FAT component shown in hot colormap. A set of labels and arrows point to specific examples where the aFAT and FAT components can be clearly identified.

A graphical representation for illustrative purposes of the significant clusters detected by the VBM procedures with regards to language and working memory performance can be seen in both Figure 2 (axial slices) and Figure 3 (sagittal slices).

**FIGURE 4:**
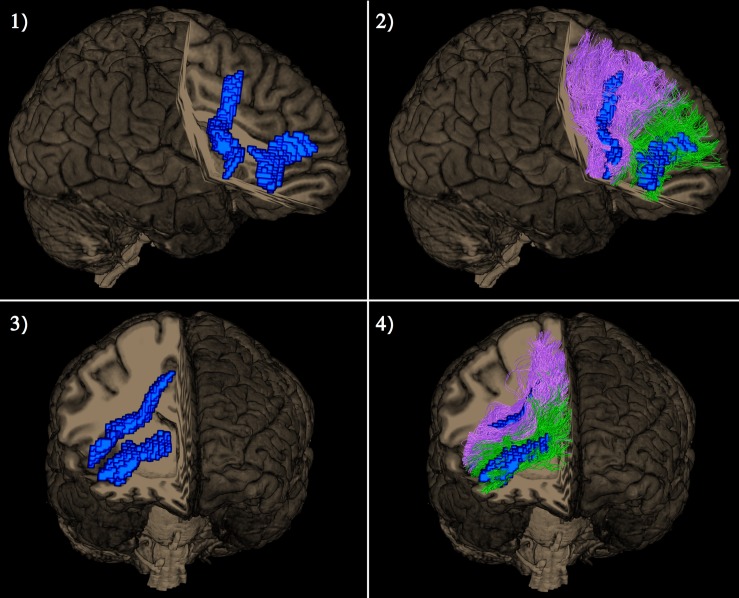
Lateral view (pictures 1 and 2 in the upper row) and frontal view (pictures 3 and 4 in the lower row) of a single subject representation of distinct right extended frontal aslant components as captured by the tractography-based VBM procedure. The significant clusters are shown in blue. Posterior vertical exFAT fibers, shown in purple, correspond exclusively to the language exFAT component. Anterior exFAT fibers (aFAT), shown in green, correspond exclusively to the working memory exFAT component.

A single subject representation of the functionally distinct right exFAT components as detected by the tractography-informed VBM procedure can be seen in Figure 4.

## Discussion

### The Need of Tract-Specific Procedures

Inferences based on lambda parameters alone must be done with caution, as evidence suggests that these parameters properly explain WM pathology only in presence of simple fiber architecture, or in very homogeneous fiber systems (De Santis et al., 2014), while in more complex situations, such as with the presence of crossing fibers, it is not easy to separate variations in lambda parameters attributable to pathology, crossing fibers and partial volume effects caused by residual misalignment. For this reason, a strong motivation behind this work was to develop methods capable of leveraging tract-specific information present in high quality datasets to help overcome this limitation.

### Hypotheses Validation

Hypothesis 1 was partially validated by detecting a right hemisphere FAT component in the maximum variation sampling by language performance group whose average fiber density was significantly correlated with language performance, but failing to detect a significant left hemisphere FAT component.

Hypothesis 2 was validated by detecting a right hemisphere aFAT component in the maximum variation sampling by working memory performance group, whose average fiber density was significantly correlated with working memory performance.

Hypothesis 3 was validated by detecting only a right hemisphere aFAT component in the maximum variation sampling by working memory performance group, and failing to detect any left-hemisphere component.

### Diffusion-Derived Microstructural Parameter Analyses

The results here presented add to the well-established evidence of the implication of the FAT in language function, as detected by the VBM analyses performed both on diffusion parameters (AD, RD, MD, and FA) and on fiber density (both territory-informed and tractography-informed). In the case of the studies based on diffusion parameters, only the FA analysis was capable of detecting an anterior exFAT component, while the analysis of the lambda diffusivity parameters (AD, RD, and MD) did not show any significant result related to working memory performance. This anterior cluster detected in the FA analysis was quite small compared with every other reported cluster.

### Fiber Density-Based Analyses

The territory-informed VBM analysis in the fiber density study was capable of detecting a larger anterior exFAT component compared to the FA study, and crucially, the tractography-informed procedure showed the presence of a cluster with ∼6x the number of voxels of the territory-informed results (and ∼30x the number of voxels of the aforementioned FA result). The presented results show that both diffusion parameter-based and fiber density-based studies were able to detect significant clusters with similar spatial coordinates, pointing to the presence of a common structural difference, and in addition to this, the results related to the anterior exFAT component indicate that the methodology here presented can be used to successfully exploit the anatomical information present in the advanced tractography reconstructions and thus detect differences in situations where they are not apparent by using more traditional measurement of diffusion parameters.

### Inter-Hemispheric Differences in White Matter Microstructure

Contrary to what was expected as per Hypothesis 1, the presented methodology failed to detect a significant language-related FAT component in the left exFAT territory. Although the human brain is a roughly bilaterally symmetrical structure, it is known to present a number of structural inter-hemispherical asymmetries underlying its functional lateralization. Such differences have been reported in language-related cortical areas in a multi-modal parcelation study (Glasser et al., 2016), is present in a number of well-studied tracts (Thiebaut de Schotten et al., 2011), and has been specifically reported in language-related pathways (Powell et al., 2006). Thus, it is conceivable that while parameters such as tract volume, which could behave similarly in the bilateral frontal aslant connections, inter-hemispheric differences in white matter microstructure could explain this asymmetry in the presented results. Other complementary studies should be performed to better characterize the structural implications of the left exFAT territory in brain functions.

### A Common Neural Substrate for Language and Working Memory

It has been posited that language and working memory function could share a common neural substrate where Broca’s area (Koechlin and Jubault, 2006) and the dorsal region of the frontal cortex (Badre and D’Esposito, 2009) exhibit an anteroposterior gradient where more anterior regions are responsible for more abstract processing. Furthermore, this property has been proposed to be a general characteristic of the frontal cortex (Fuster, 1989). Given the strategic placement of the exFAT territory in the frontal lobe, the presented results support the interpretation that, in the right hemisphere, distinct exFAT components exist along said anteroposterior gradient, with a posterior component mediating language performance (the FAT) and an anterior component mediating more abstract working memory capacity (the aFAT). This evidence suggests that the exFAT could function as a common neural interface where language and working memory function are integrated in the healthy human brain.

### VBM Modulation and Fiber Density

We decided to apply Jacobian modulation as part of our VBM pipeline for analyzing differences along the exFAT white matter territory under the interpretation that fiber density is an indirect measure of the “quantity” of connectivity between regions which should be preserved under spatial transformations. Thus regions that are contracted during transformation should be interpreted as fibers being more densely packed in a smaller volume, while the opposite should be true for small regions being expanded. Interpreting unmodulated images would lead to incorrectly detecting differences in cases where the same “quantity” of connectivity (i.e., fiber count) is present within differently-sized volumes.

### Choice of Kernel Size

The usage of larger kernel sizes increases tolerance against fine misregistration errors and improves sensitivity at the expense of degraded specificity. In the white matter territory, it has been noted that smoothing can increase the uncertainty of the position of peak voxels, leading to artifactual bridging of independent regions (Reimold et al., 2006). In this study we wanted be sensitive to potentially small but distinct regions of local difference by leveraging the high-quality data of the HCP sample. To this end, we opted for a conservative kernel size of 2 mm. We reason that in a 3D white matter structure residual misregistration is a more forgiving problem compared to the gray matter situation, where regions are often completely disjoint among subjects upon registration due to common anatomical variations and other inter-subject structural differences of a highly convoluted 2D cortical sheet folded in 3D space, and thus, in the present situation, the usage of kernel sizes commonly employed for VBM studies of gray matter territories (8–16 mm) would further blur the images and increase the likelihood of detecting spatially displaced and wrongfully merged results.

### Limitations and Future Work

It must be noted that the Binder language task was designed to elicit strong activation of the temporal lobe for pre-surgical assessment of epilepsy patients, and subjects present a strong ceiling effect in its scores. Thus, using a more discriminative language task could result in an increased statistical power in the detection of differences attributable to language capacity performance differences among high performers.

In the context of a maximum variation sampling study, results must be interpreted with care: given that we are detecting differences between two extremes of the sample, we cannot directly infer the existence of an anatomical ground truth consisting of an independent anterior frontal aslant tract in the general population, but we can support the hypothesis that the exFAT territory structure varies differentially along its anteroposterior axis according to separate cognitive abilities. Further studies should be performed in order to elucidate whether the aFAT component is better described as a separate white matter tract, or as a functionally and anatomically distinct subregion of a wider exFAT territory.

Given that inter-hemispherical differences in white matter microstructure could render local differences in fiber density a poor parameter for probing the left exFAT territory, further studies with alternative techniques should be conducted to determine whether a contralateral homolog of the right aFAT can be detected, as well as to determine its potential implication in different cognitive functions.

## Conclusion

We performed tractography-informed VBM analyses on fiber density along the bilateral exFAT territory in two maximum variation sampling studies by comparing top performers and bottom performers in language and working memory tasks selected from the HCP sample. This study was able to recover, without prior constraints, an approximation of the original right FAT related to language performance. Additionally, we detected the presence of a distinct cluster related to working memory performance corresponding to a novel right anterior frontal aslant component (the aFAT).

The reported results point to the right exFAT territory as a candidate structural scaffold where language function and working memory interface via a common neural circuit. This finding is compatible with previously reported data on the hierarchical structural organization of both Broca’s region and the frontal cortex along an anteroposterior axis, and opens the door to better informed surgical approaches to the frontal lobe. Additionally, the presented results can contribute to improve theoretical models by providing a structural basis accounting for an integrative view of language and working memory function in the human brain, leading to better clinical understanding of language and working memory alterations in the human brain.

## Data Availability Statement

The datasets generated for this study are available on request to the corresponding author.

## Ethics Statement

The studies involving human participants were reviewed and approved by Bioethics Committee of the University of Barcelona [Institutional Review Board (IRB00003099)]. The patients/participants provided their written informed consent to participate in this study.

## Author Contributions

FV and SP-D discussed and conceived the idea, designed and implemented the necessary software for the study, verified the results and contributed equally to the writing of the final manuscript. AP-G supervised the work and contributed to the revision of the manuscript.

## Conflict of Interest

The authors declare that the research was conducted in the absence of any commercial or financial relationships that could be construed as a potential conflict of interest.
