# Supplementary material for: Distinct Components in the Right Extended Frontal Aslant Tract Mediate Language and Working Memory Performance: A Tractography-Informed VBM Study
Source: Front Neuroanat. 2020 Apr 21;14:21. doi: 10.3389/fnana.2020.00021 (PMC7186483; doi:10.3389/fnana.2020.00021)
Supplement: Supplementary file 1 [file Data_Sheet_1.DOCX]

Supplementary Material

## Supplementary Figures


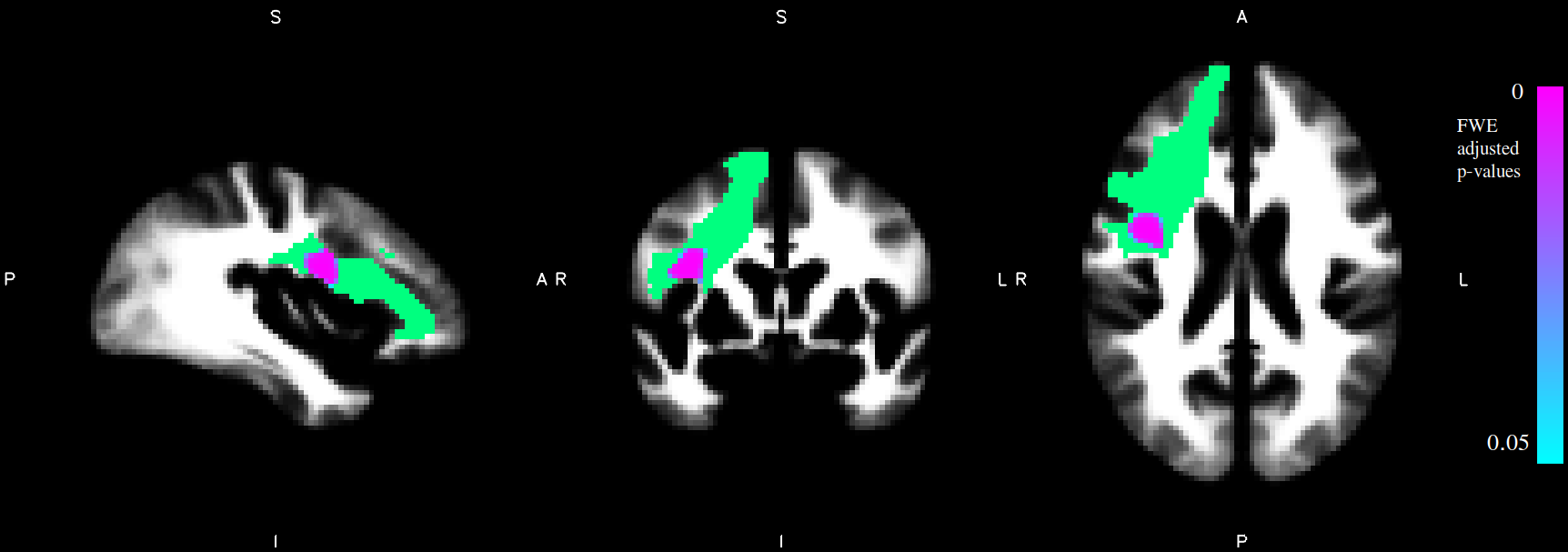


**Supplementary Figure 1.** AD clusters for correlations VBM: right exFAT – Language. The cluster component shown in cool colormap indicates significant voxels forming a posterior component in the correlations VBM analysis of AD maps in the language study.


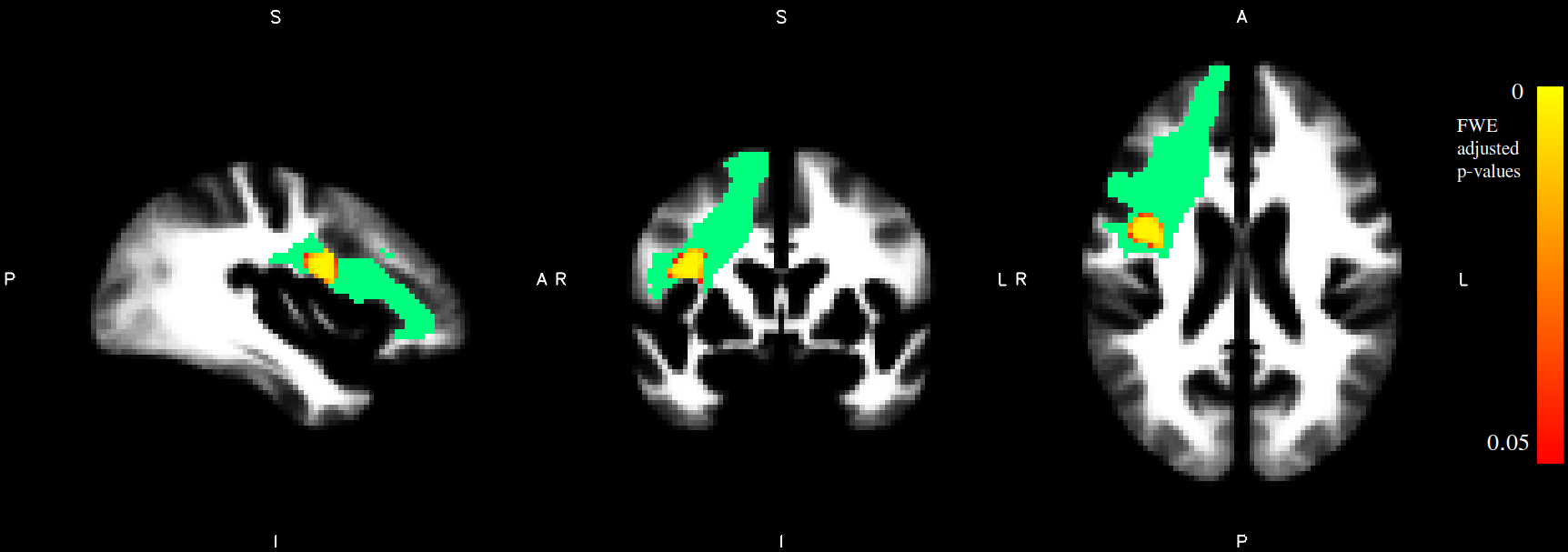


**Supplementary Figure 2.** AD cluster for t-tests VBM: right exFAT – Language. The cluster component shown in hot colormap indicates significant voxels forming a posterior component in the t-tests VBM analysis of AD maps in the language study.

*
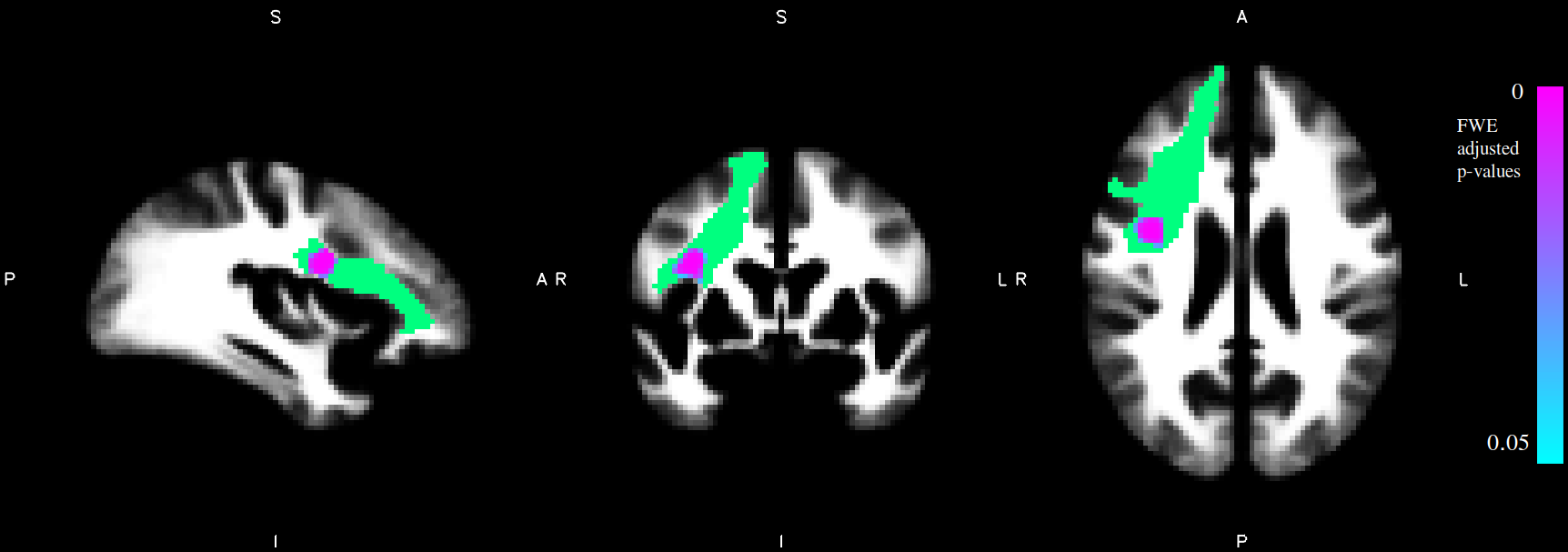
*

**Supplementary Figure 3.** RD cluster for correlations VBM: right exFAT – Language. The cluster component shown in cool colormap indicates significant voxels forming a posterior component in the correlations VBM analysis of RD maps in the language study.


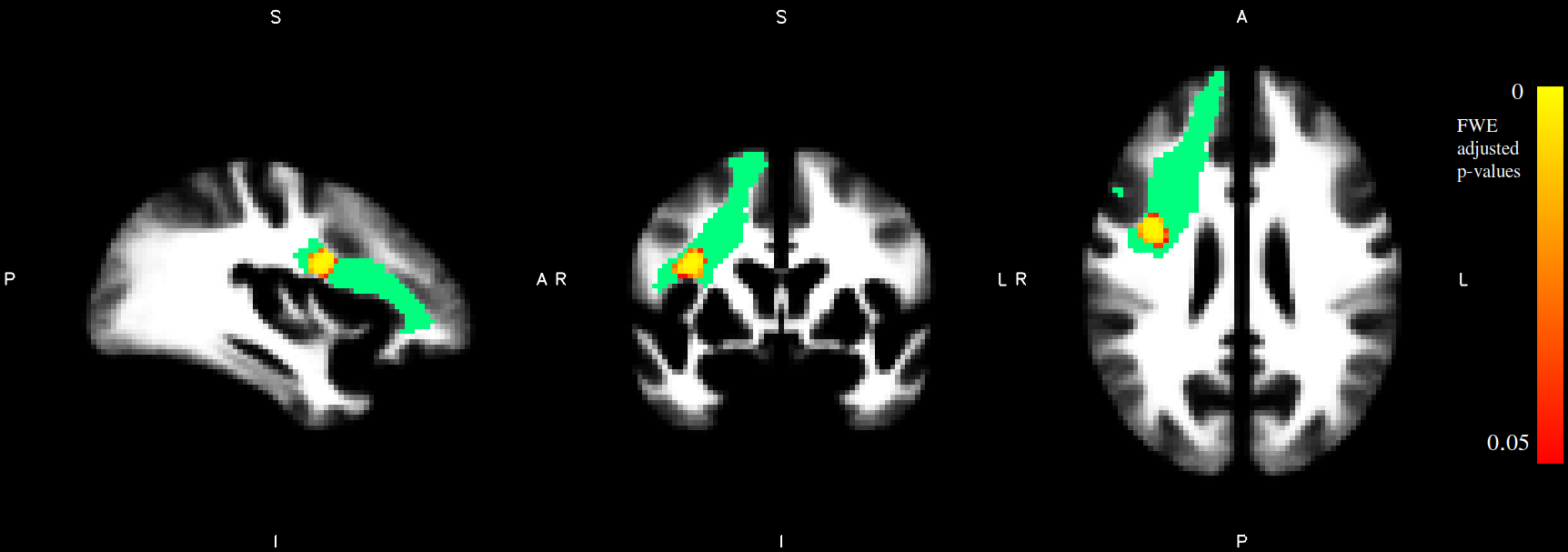


**Supplementary Figure 4.** RD cluster for t-tests VBM: right exFAT – Language. The cluster component shown in hot colormap indicates significant voxels forming a posterior component in the t-tests VBM analysis of RD maps in the language study.

*
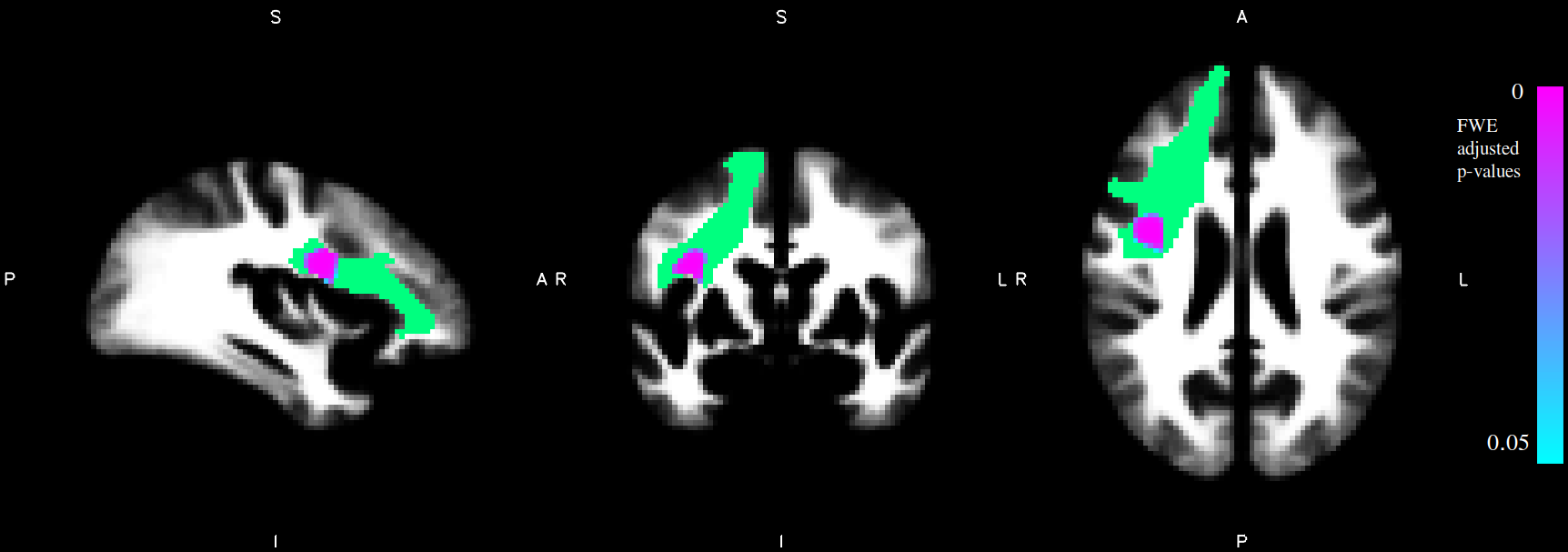
*

**Supplementary Figure 5.** MD cluster for correlations VBM: right exFAT – Language. The cluster component shown in cool colormap indicates significant voxels forming a posterior component in the correlations VBM analysis of MD maps in the language study.


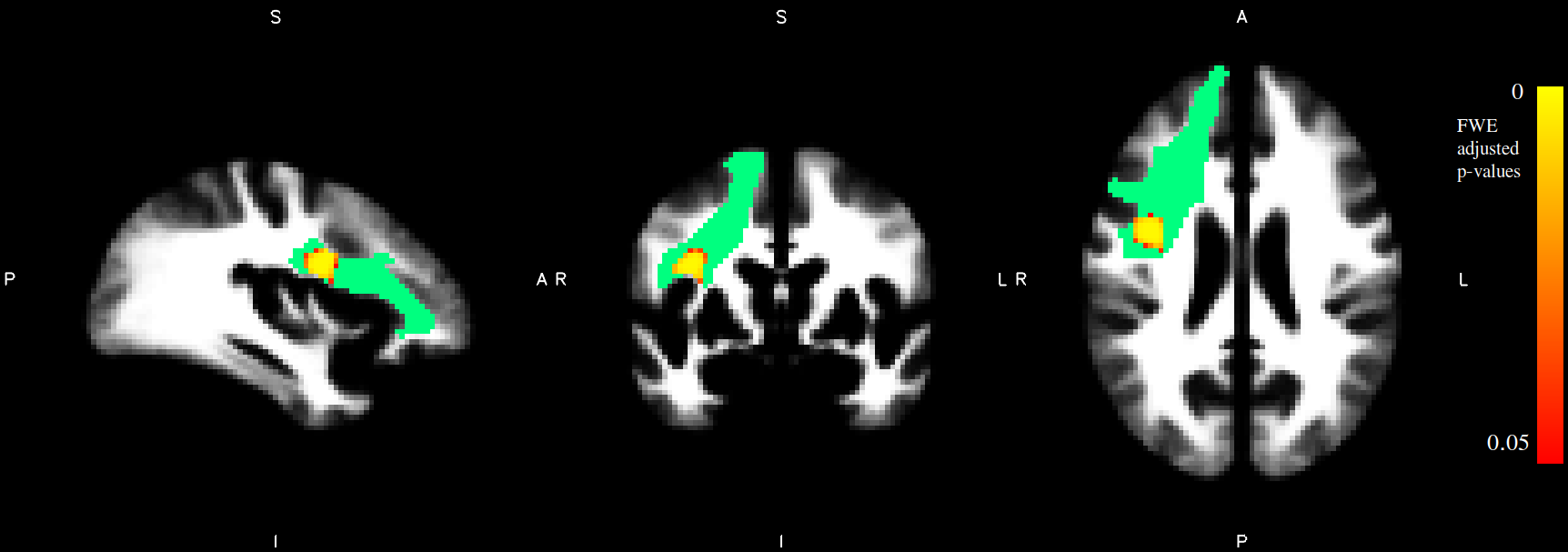


**Supplementary Figure 6.** MD cluster for t-tests VBM analysis of right exFAT – Language. The cluster component shown in hot colormap indicates significant voxels forming a posterior component in the t-tests VBM analysis of MD maps in the language study.

*
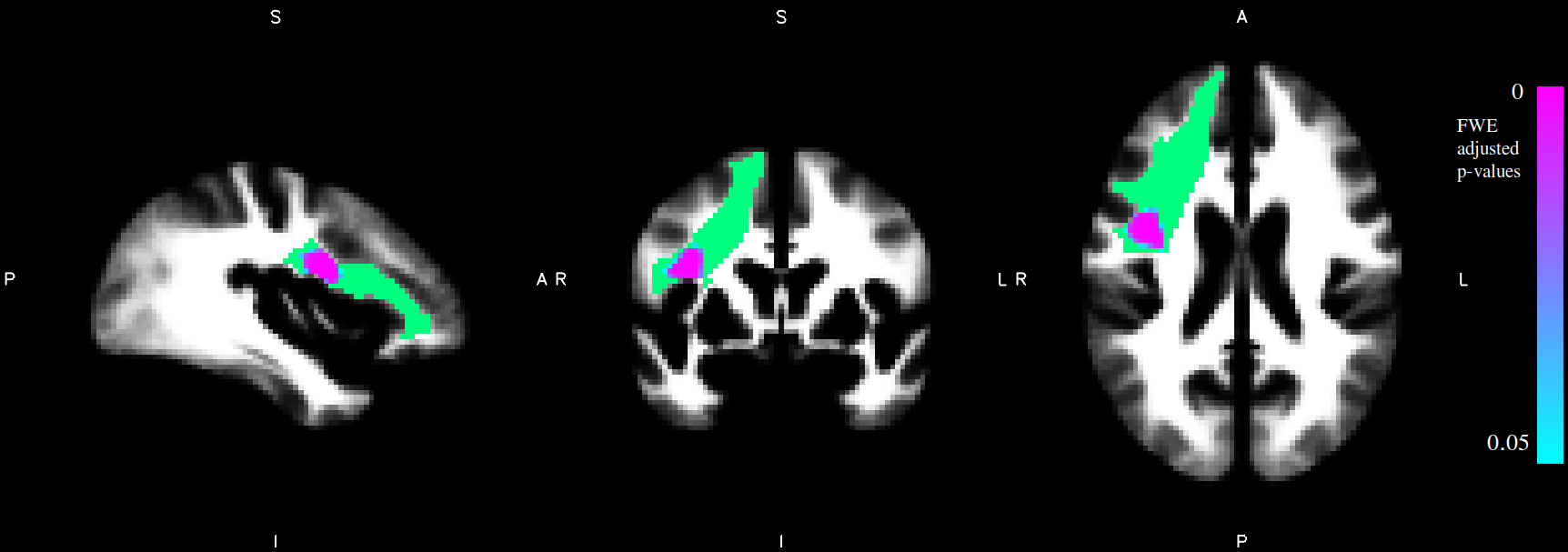
*

**Supplementary Figure 7.** FA cluster for correlations VBM: right exFAT – Language. The cluster component shown in cool colormap indicates significant voxels forming a posterior component in the correlations VBM analysis of FA maps in the language study.


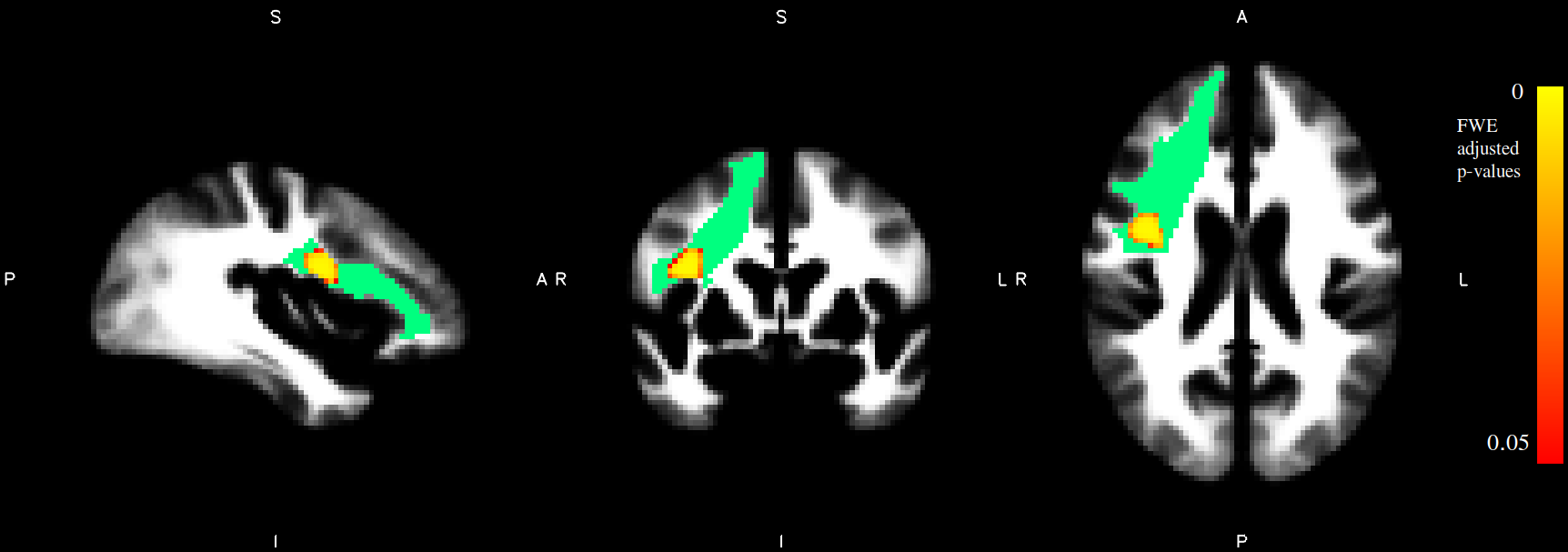


**Supplementary Figure 8.** FA cluster for t-tests VBM: right exFAT – Language. The cluster component shown in hot colormap indicates significant voxels forming a posterior component in the t-tests VBM analysis of FA maps in the language study.


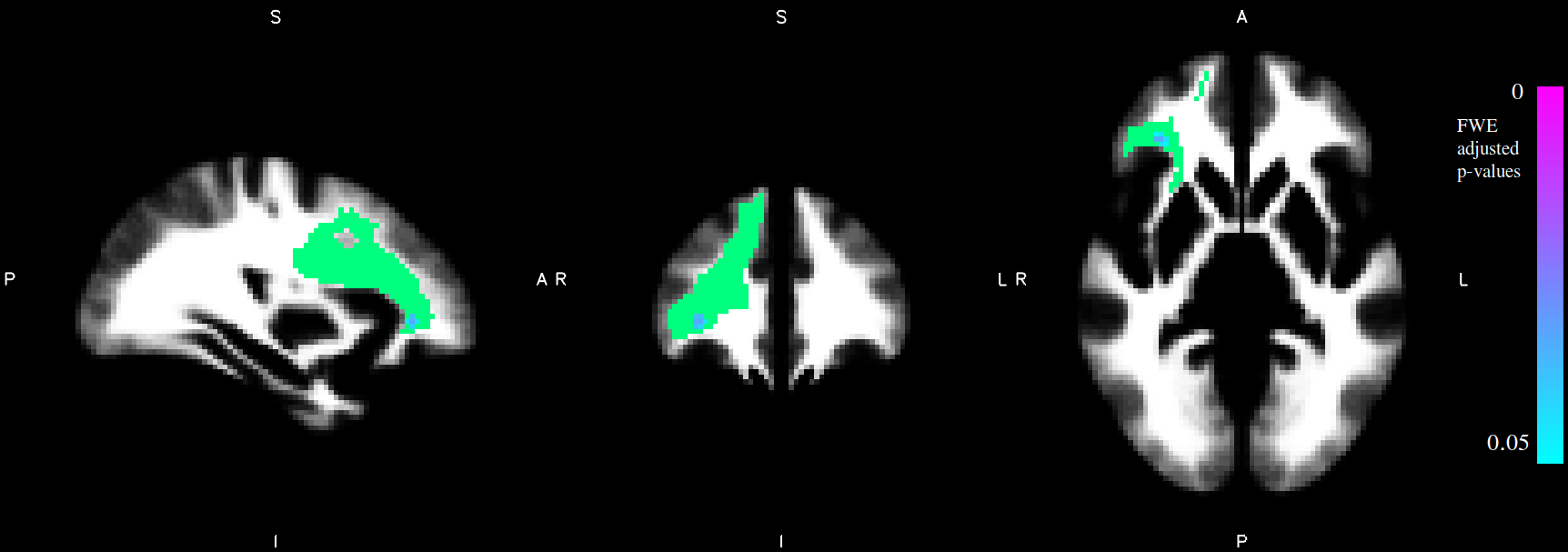


**Supplementary Figure 9.** FA cluster for correlations VBM: right exFAT - Working Memory. The cluster component shown in cool colormap indicates significant voxels forming an anterior component in the correlations VBM analysis of FA maps in the working memory study.
